# Integrative bioinformatic analyses of an oncogenomic profile reveal the biology of endometrial cancer and guide drug discovery

**DOI:** 10.18632/oncotarget.6716

**Published:** 2015-12-22

**Authors:** Henry Sung-Ching Wong, Yung-Shun Juan, Mei-Shin Wu, Yan-Feng Zhang, Yu-Wen Hsu, Huang-Hui Chen, Wei-Min Liu, Wei-Chiao Chang

**Affiliations:** ^1^ Master Program for Clinical Pharmacogenomics and Pharmacoproteomics, School of Pharmacy, Taipei Medical University, Taipei, Taiwan; ^2^ Department of Clinical Pharmacy, School of Pharmacy, Taipei Medical University, Taipei, Taiwan; ^3^ Department of Urology, Kaohsiung Municipal Hsiao-Kang Hospital, Kaohsiung, Taiwan; ^4^ Department of Urology, College of Medicine, Kaohsiung Medical University, Kaohsiung, Taiwan; ^5^ HudsonAlpha Institute for Biotechnology, Huntsville, AL, USA; ^6^ The Ph.D. Program for Translational Medicine, College of Medical Science and Technology, Taipei Medical University, Taipei, Taiwan; ^7^ Academia Sinica, Taipei, Taiwan; ^8^ Department of Obstetrics and Gynecology, School of Medicine, Taipei Medical University, Taipei, Taiwan; ^9^ Department of Obstetrics and Gynecology, Taipei Medical University Hospital, Taipei, Taiwan; ^10^ Department of Pharmacy, Taipei Medical University-Wan Fang Hospital, Taipei, Taiwan; ^11^ Center for Biomarkers and Biotech Drugs, Kaohsiung Medical University, Kaohsiung, Taiwan

**Keywords:** endometrial cancers, expression-associated somatic mutations, cancer genomics, cancer drivers, cancer drug repurposing

## Abstract

A major challenge in personalized cancer medicine is to establish a systematic approach to translate huge oncogenomic datasets to clinical situations and facilitate drug discovery for cancers such as endometrial carcinoma. We performed a genome-wide somatic mutation-expression association study in a total of 219 endometrial cancer patients from TCGA database, by evaluating the correlation between ∼5,800 somatic mutations to ∼13,500 gene expression levels (in total, ∼78, 500, 000 pairs). A bioinformatics pipeline was devised to identify expression-associated single nucleotide variations (eSNVs) which are crucial for endometrial cancer progression and patient prognoses. We further prioritized 394 biologically risky mutational candidates which mapped to 275 gene loci and demonstrated that these genes collaborated with expression features were significantly enriched in targets of drugs approved for solid tumors, suggesting the plausibility of drug repurposing. Taken together, we integrated a fundamental endometrial cancer genomic profile into clinical circumstances, further shedding light for clinical implementation of genomic-based therapies and guidance for drug discovery.

## INTRODUCTION

In the United States, uterine corpus endometrial carcinoma (UCEC) or endometrial cancer is the third most common malignancy and the most prevalent genital-system tumor among females, with an estimated 54,870 new cases and 10,170 deaths in 2015 [1]. Endometrial cancer transformation arises from the normal uterine corpus through a progressive accumulation of somatic and epigenetic aberrations.

On the basis of disease stage, early-stage endometrial cancer can generally be cured, while recurrent and advanced endometrial cancer is clinically aggressive, and progress in developing treatment approaches has been limited [2]. The dearth of new clinical drugs has led to a new approach called drug repurposing, the alleged new uses for old drugs, to increase usable therapeutic agent access for endometrial cancers. The rationality of drug repurposing of approved anticancer drugs is based on the concept that different cancers may possess similar pathological molecular or pathway origins. The most noticeable feature of drug repurposing is the clear understanding of pharmacological mechanisms, pharmacokinetic profiles, metabolism pathways and toxic reactions of approved drugs [3]. In estimation, approximately 90% of old drugs can be developed for secondary purposes [4], further provide evidence for the feasibility of drug repurposing as a drug discovery tool.

The Cancer Genome Atlas (TCGA) database is a large-scale genomic database that primarily envisions somatic changes of cancers including endometrial cancer [5]. The final goal of these tremendous efforts is to tailor patient treatment in a personalized manner, and drive customization of disease care by implementing tumor genetic information. Importantly, the identified drivers can act as drug targets for disease amelioration, treatment biomarkers for patient categorization, and prognostic markers for patient survival. Although the landscape of somatic aberrations in endometrial cancer has been depicted, the potential utilization of relevant biomarkers into clinical situations has been limited. Moreover, studying large amount of cancer genomic data remains analytically challenging. The genetic architecture of transcription profiles is thoroughly affected by several kinds of genetic and epigenetic polymorphisms [6, 7]. Consequently, the presence of other genetic determinants may increase the difficulty in clarifying the effects of somatic mutations on gene expressions.

Taken together, the above-described situations motivated this integrative study to determine expression-associated somatic alterations and quantify their aberrations on the cancer transcriptome. These expression-associated (e)SNVs were further prioritized by a scoring system based on 7 criteria to identify biologically risky mutational candidates and provide crucial information for drug discovery.

## RESULTS

### Identification of eSNVs in endometrial cancer

**Figure 1:**
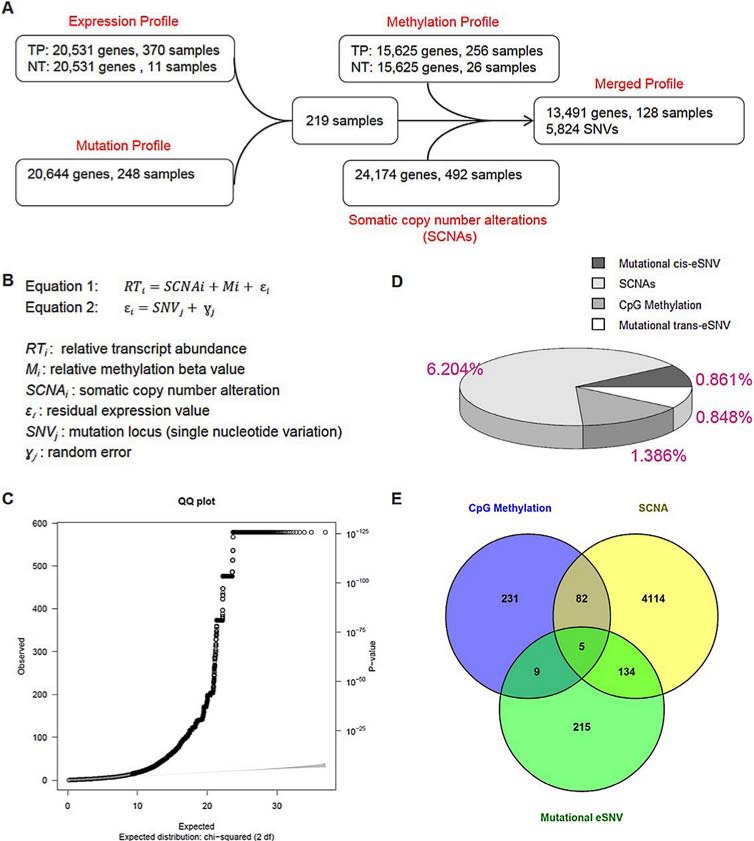
Correlation between somatic mutations and transcripts in endometrial cancers **A.** Schematic showing filtering procedures of the expression, mutation, methylation, and somatic copy number alteration profiles. Expression data of primary tumors from transcription sequencing were normalized by expression values of available adjacent normal tissues. Mutated genes extracted from DNA sequencing underwent data sanitization before being intersected with expression data. In total, we retrieved 219 endometrial cancer samples for analyses. To conduct the multivariate linear regression analysis, gene methylation data (normalized by beta values from available normal adjacent tissues) and somatic copy number data were filtered against 219 samples, resulting in a merged profile that included 128 samples with 13,491 gene profiles and 5,824 somatic mutations. Expression-associated single-nucleotide variations were detected by the merged profile and further explored in 219 samples. **B.** Equation for the multivariate linear regression. In Equation 1, residual relative transcript abundance values of methylation levels and somatic copy number alterations were further correlated with single-nucleotide-based somatic mutation statuses as shown in Equation 2. **C.** To evaluate overdispersion and other sources of bias or confounding, a quantile-quantile plot was drawn for single-nucleotide variant (SNV) analytical results of all 13,491 genes. A genomic control inflation factor (λ value = 0.91) was calculated by the ratio of the observed mean to the corresponding expected value. **D.** A pie chart showing the relative (area) and absolute (percentage labeled) fraction of the variance explained by 3 determinants: SCNAs, CpG methylation and SNVs. The effects of *cis*-acting and *trans*-acting SNVs were separately estimated. **E.** Venn diagram showing the number of genes under regulation of 3 determinants: SCNAs [yellow], CpG methylation [blue] and SNVs [green].

We applied a 2-step linear regression to correlate the somatic mutation status with the residual expression value while adjusting for 2 genetic determinants (Figure 1B). Using Equations 1 and 2 (Supplementary Figure 1D), we identified 74,713 significant SNV-gene pairs (0.095% from the total 78,571, 584 SNV-gene pairs), which mapped to 4,153 eSNVs (71.3%) and 2,612 unique genes (19.4%). A Q-Q plot of all raw *p* values revealed a low possibility of sample relatedness (λ_GC_ = 0.91, Figure 1C). The effects of mutational eSNVs were estimated and these respectively revealed 0.861% and 0.848% explanations of the mean total variation of the residual relative transcript abundance by *cis*- and *trans*-acting eSNVs (Figure 1D).

We identified expression levels of 4,335 genes (32.13% of all genes) that were significantly correlated with their somatic copy number (with an FDR of < 0.05), which accounted for 6.204% of the mean total variation of expression levels. Respectively, expression levels of 327 genes (2.42% of all genes) were significantly correlated with methylation of CpG islands (with an FDR of < 0.05), which explained 1.386% of the mean of total variation of expression levels (Figure 1D). Among those genes that showed correlations of their expression values with both the somatic copy number and methylation, 5 (*ERBB2, PDXK, HDAC4, PLXNA1* and *TNFAIP2*) were also correlated with mutational eSNVs (Figure 1E).

### Gene-based prioritization and mutational cluster identification

**Figure 2:**
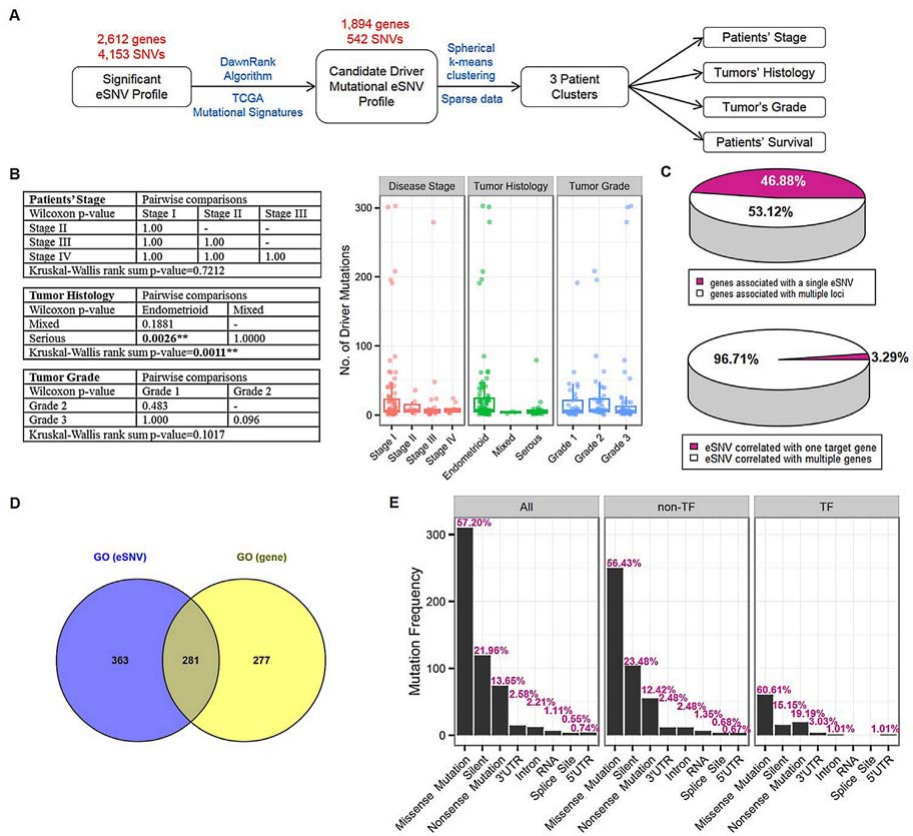
Gene-based prioritization and mutational cluster identification **A.** Schematic showing prioritization procedures and a subsequent cluster identification analysis. Significant expression-associated single-nucleotide variations (eSNVs) that met the Bonferroni criteria were further prioritized by candidate driver mutations identified by the *DawnRank* algorithm and TCGA mutational signatures. The resulting 542 eSNVs underwent spherical k-means clustering in 219 endometrial cancer samples. To elucidate the contribution of patients’ mutation profiles to clinical statuses (disease stage, tumor histology, tumor grade, and survival), correlation analyses were conducted using 3 identified mutational clusters. **B.** Correlation between number of driver mutations and patients clinical profiles. Kruskal-Wallis rank sum tests followed by pairwise Wilcoxon tests with Bonferroni correction were carried out to elucidate the correlation between driver gene number and patients’ disease stage, tumor histology and tumor grade [left]. As a result, patients with endometrioid and serious-type endometrial cancer showed significant enrichment in driver mutations (pairwise Wilcoxon *p*-value [Bonferroni-adjusted] = 0.0026). Box plots showed the distribution pattern of driver mutations in each subcategory of patients’ clinical profiles [right]. **C.** Pie charts showing proportions of 1,894 genes associated with single or multiple eSNV(s) [top] and 542 eSNVs correlated with transcript levels of single or multiple genes [bottom]. **D.** Venn diagram showing significant over-represented gene ontology terms by 357 genes mapped from 542 eSNVs [blue], and 1,894 genes with expression levels correlated with identified eSNVs [yellow]. **E.** Bar plots showing frequency distributions of mutation types in all 542 eSNVs (“All”), 443 eSNVs that did not map to transcription function-related genes (“non-TF”), and 99 eSNVs that mapped to genes annotated to be transcription function-related (“TF”). Corresponding relative frequencies of each mutation type are labeled. **P < 0.01.

We distinguished driver mutations from functionally neutral passenger mutations with the *DawnRank* algorithm, and included prior-identified genes from TCGA in the prioritization step. As a result, a gene list containing 1,619 genes was generated for endometrial cancer (Figure 2A and Supplementary Figure 2A). The number of driver mutations showed a significant association with the tumor histology (Kruskal-Wallis rank sum *p* = 0.0011, Supplementary Figure 2B), but not disease stage (Kruskal-Wallis rank sum *p* = 0.7212) or tumor grade (Kruskal-Wallis rank sum *p* = 0.1017). Pairwise comparisons revealed significant difference between borne driver mutations of the endometrioid subtype compared to serous-like endometrial cancer (Bonferroni-adjusted Wilcoxon *p* = 0.0026, Figure 2B).

Next, we prioritized eSNVs based on their corresponding gene according to the *DawnRank* gene list and identified 542 driver eSNVs, which correlated with expression levels of 1,894 genes. This candidate driver mutational eSNV profile contained 9,488 significant associations (SNV-gene pairs). Of the 542 driver eSNVs (which mapped to 357 gene loci), 18 (3.32%) were associated with the transcript level of a single gene, and the remaining ones (96.68%) were associated with transcript levels of multiple genes. Of the 1894 unique genes, 888 genes (46.88%) were correlated with the mutational status of a single driver eSNV, while the remaining ones (53.12%) were correlated with the mutation statuses of multiple driver eSNVs (Figure 2C). Among all prioritized driver eSNVs, 3 were *cis*-acting mutational loci (Table 1), which were separately located on *ATF7IP* (chr12:14576892 T > C), *TP53* (chr17:7578271 C > T), and *XPO7* (chr8:21827087 C > T). These 3 *cis*-acting eSNVs were also correlated with transcript levels of another 169 genes. The other 539 *trans*-acting driver mutational eSNVs significantly correlated with the relative transcript abundances of 1894 genes (Supplementary Table 1).

**Table 1:** Prioritized cis-acting expression-associated single-nucleotide variations (SNVs)

Chr:Pos	SNV	Gene locus	*β* Coef	95% CI	*p* value	VarExplained
12:14576892	12:14576892 C > T	*ATF7IP*	0.607	0.48–0.74	7.87E-16	0.404
17:7578271	17:7578271 T > C	*TP53*	2.29	1.72–2.86	9.94E-13	0.333
8:21827087	8:21827087 C > T	*XPO7*	0.78	0.57–0.99	1.32E-11	0.306

Chr:Pos: Chromosome:Position.

SNV: SNVs lacking a reference single-nucleotide polymorphism number are illustrated by Chr:Pos. The allele change in the primary tumor is shown.

Gene locus: Gene locus where the SNV is located.

β Coef: Beta-coefficient calculated from a linear regression correlation analysis.

95%CI: 95% confidence interval.

*p* value: Raw *p* value calculated from a linear regression correlation analysis.

VarExplained: Explanation of the mean total variation (*r*^2^) of the residual relative expression of the corresponding gene.

We further conducted GO enrichment analysis (Figure 2D) and transcription factor-related annotation (Figure 2E) based on AnimalTFDB to explore the 542 prioritized eSNVs. The ORA revealed 645 significantly enriched GO terms in biological processes, particular in cellular signaling, cellular apoptosis, cellular differentiation, and cellular proliferation processes, indicating the extensive dysregulation of cellular processes in endometrial cancer due to somatic alterations (Supplementary Figure 2C and Supplementary Table 2). In addition, an enrichment analysis based on 1,894 correlated genes also provided over-representative evidence of 559 biological processes. Further inquiry of 2 significant GO lists revealed 281 intersecting biological processes (hypergeometric test *p* < 0.001, Figure 2D). We further explored 542 candidate driver eSNVs by combining information from AnimalTFDB (Supplementary Figure 2D). As a result, we identified 50 genes mapped by 99 eSNVs that were TF-related (Supplementary Figure 2E). We further assessed mutation type proportions in all eSNVs, the TF-related group, and the non-TF-related group (Figure 2E). The mutation type proportion of the non-TF-related group was similar to the distribution of all 542 eSNVs, and the top 3 most enriched mutation types in decreasing order were missense (56.43%, multiple of change (MC) = 0.93), silent (23.48%, MC = 1.09), and nonsense (12.42%, MC = 0.90). Depiction of the distribution of the TF-related group disclosed a slightly different pattern than that of the non-TF-related group, which the top 3 enriched mutation types in decreasing order were missense (60.61%, MC = 1.15), nonsense (19.19%, MC = 0.64) and silent (15.15%, MC = 1.5). However, no significant difference in distribution patterns across TF-related and non-TF-related groups was detected based on the hypergeometric test (all *p* values > 0.05).

**Figure 3:**
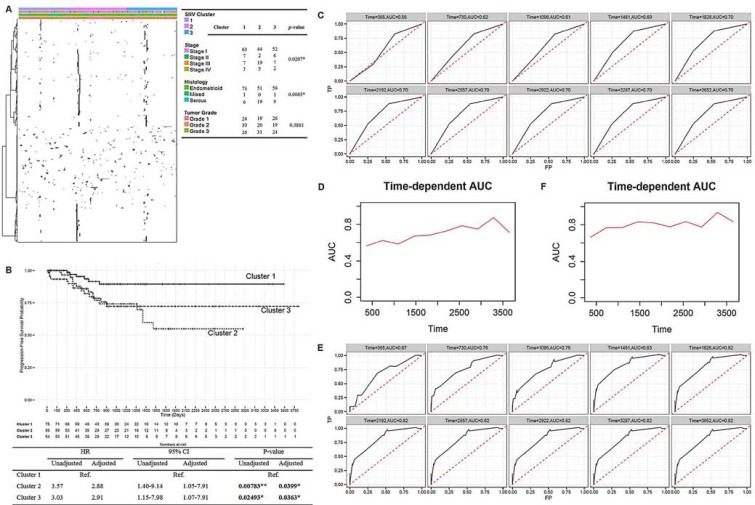
Mutation signatures of endometrial cancers and its prognostic potential **A.** Heatmap of 542 eSNVs revealed the sparse nature of the mutation profiles. Patients were sorted into 3 identified mutational clusters. Sample clinical profiles against 3 clusters are shown, and Fisher's exact test was used to evaluate the significance. **B.** Kaplan-Meier plot [top] showing progression-free survival curves of endometrial cancer patients in the 3 mutational clusters. Summary statistics are shown in the table [bottom]. **C., E.**. Time-dependent receiver operating characteristic curves and cumulative/dynamic areas under the curve of identified mutation clusters without (C) or with adjustment (E) for clinical profiles. **D., F.** Cumulative case/dynamic control AUC of three identified mutational clusters were calculated by Uno's method to assess the predictive accuracy of mutation profile to progression-free survival in a 10-year time-dependent manner without adjustment (D) or with adjustment (F) *P < 0.05, **P < 0.01.

We then conducted an unsupervised spherical k-means clustering to classify endometrial cancer patients (*n* = 219) based on their mutation profile of 542 driver eSNVs into 3 main clusters, denoted clusters 1, 2, and 3 (Figure 3A). The identified mutational clusters showed significant correlations with endometrial cancer patients’ clinical profiles, including disease stage (Fisher's exact *p* = 0.0207) and tumor histology (Fisher's exact *p* = 0.0065), but not tumor grade (Fisher's exact *p* = 0.3861). Based on these results, cluster 2 showed significant enrichment in late-stage patients (stages III and IV, 24 in cluster 2 vs. 10 and 9 in clusters 1 and 3, respectively) and serous-like endometrial cancers (19 in cluster 2 vs. 6 and 9 in clusters 1 and 3, respectively), which was correlated with a poor prognosis in patients.

We also observed a significant progression-free survival-related characteristic of mutational clusters (Figure 3B). However, the identified clusters showed no association with the overall survival of patients (data not shown). Patients in clusters 2 and 3 showed increase risks of death compared to patients in cluster 1 (hazard ratio (HR)_2vs1_ = 3.57 and 95% confidence interval (CI)_2vs1_ = 1.40∼9.14, *p*_2vs1_ = 0.00783). Similarly, cluster 3 was associated with a poorer prognosis compared to cluster 1 (HR_3vs1_ = 3.03, 95% CI_3vs1_ = 1.15∼7.98, *p*_3vs1_ = 0.02493). As mutational clusters were also associated with disease aggressiveness, these factors were further adjusted for to avoid biased estimations of the effects of the identified clusters on the prognostic potential. Consistently, mutational clusters showed an association after adjusting for clinical profiles, with cluster 1 showing a significantly better prognosis (adjusted *p*_2vs1_ = 0.0399; adjusted *p*_3vs1_ = 0.0363) compared to the other 2 clusters (adjusted HR_2vs1_ = 2.88; adjusted HR_3vs1_ = = 2.91, adjusted 95% CI_2vs1_ = 1.05∼7.91; adjusted 95% CI_3vs1_ = 1.07∼7.91).

We then assessed the concordance between the identified mutational clusters and survival in a time-dependent manner. Before adjustment, the 10-year time-dependent ROC curve revealed that the prediction accuracy of mutational clusters increased with year, which ranged 56%∼70% (Figure 3C). In addition, the 10-year time-dependent AUC curve calculated by Uno's method also revealed a sequentially increasing pattern by year, with a summary measure of the concordance index equal to 0.6175 (Figure 3D). Intriguingly, after adjusting for the disease stage and tumor histology, the 10-year time-dependent ROC analysis revealed a higher prediction accuracy than that without adjustment (Figure 3E), which ranged 67%∼82%. The summary measure of the concordance index calculated by Uno's method was 0.7867 (Figure 3F). Therefore, a series of analyses suggested that a prominent role of patients’ mutation profiles contributed to disease biology discovery in endometrial cancer.

### Integrative analyses for drug discovery

**Figure 4:**
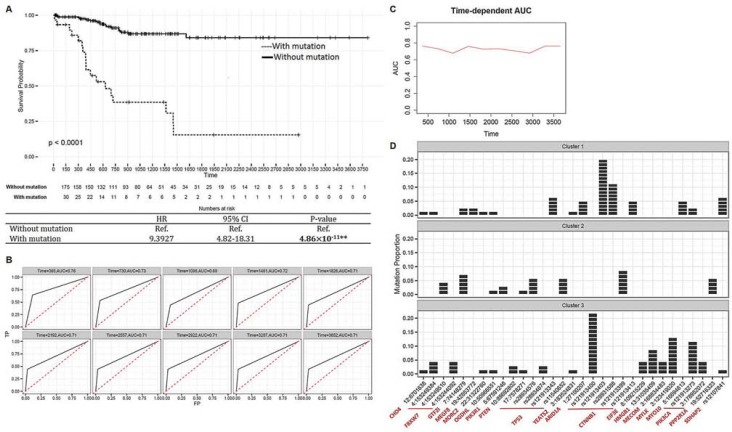
Integrative analyses for drug discovery **A.** Kaplan-Meier estimator [top] showing survival curves of endometrial cancer patients categorized according to 13 prognostic-related signatures. Summarized statistics are shown in the table [bottom]. **B.** Time-dependent receiver operating characteristic (ROC) curves of 13 prognostic-related signatures. The cumulative/dynamic the area under the ROC (AUC) curves were calculated to assess discrimination of 10-year cumulative incidences. **C.** Cumulative case/dynamic control AUC of 13 prognostic-related signatures were calculated by Uno's method to assess the predictive accuracy of mutation profile to progression-free survival in a 10-year time-dependent manner. **D.** Bar plots showing distribution of cluster-related and prognostic-related expression-associated single-nucleotide variations (eSNVs) in 3 clusters. Each patient is represented by 1 block in the bar. The x-axis represents eSNVs annotated by located genes, and the y-axis is the proportion of mutations in each cluster. **P < 0.01.

To identify biological candidate risk mutational variations in endometrial cancer patients, we conducted integrative analyses based on 542 candidate driver eSNVs and 1,894 genes the transcript levels of which were correlated with the mutational statuses of eSNVs. To identify biological candidate risk mutational variations in endometrial cancer patients, we first evaluated prognostic-related eSNV signatures using a Cox-regression. We identified 13 eSNVs which were significantly associated with prognostic-free survival in endometrial cancer patients (Supplementary Table 3). These 13 eSNVs mapped to 10 genes: *YEATS2, FBXW7, PIK3R1, GTF2I, TP53, OGDHL, PTEN, CHD4, MEGF8* and *MORC2*. We classified patients into 2 groups based on the prognostic-related signature profiles. Patients who bore at least 1 mutation in their prognostic-related signatures were compared to patients with no mutation on the 13 eSNVs. As shown, the mutation profiles of 13 eSNVs were significantly associated with progression-free survival of endometrial cancer patients (HR = 9.3927, 95% CI = 4.82∼18.31, *p* = 4.86×10^−11^, Figure 4A). A time-dependent ROC analysis revealed a summary measure of the AUC curve of 0.7365 (Figure 4B–4C). This result indicated a significant correlation between mutations and UCEC patient survival outcomes, and further confirmed the potential clinical utility of the 10 identified genes in an eSNV-based manner.

We then evaluated mutational cluster-related eSNV signatures. We identified 20 eSNVs that were significantly correlated with identified mutational clusters (Fisher's exact test < 0.05, Supplementary Figure 3A). These 20 eSNVs were mapped to 13 genes. In particular, *CTNNB1* (5 eSNVs), *PIK3CA* (2 eSNVs), and *TP53* (3 eSNVs) contained more than 1 eSNV which were cluster-related. Surprisingly, patients who bore the *CTNNB1* mutation were significantly enriched in cluster 1 compared to clusters 2 and 3 (Chi-square *p* = 2.75 × 10^−5^). Similarly, the *PIK3CA* mutation was significantly enriched in patients in cluster 3 (Chi-square *p* = 3.62 × 10^−4^). In contrast, the number of patients with the *TP53* mutation was higher in cluster 2 compared to clusters 1 and 3 (not significant, Chi-square *p* = 0.1926). Notably, our results matched previous observations that mutations of *TP53* and *PPP2R1A* were more common in the serous-like, which was enriched in cluster 2. Comparatively, mutations of *PTEN* and *ARID1A* were more common in endometrioid endometrial cancer, which was enriched in clusters 1 and 3. Cluster-related and prognostic-related signatures were combined and are illustrated with mutational clusters (Figure 4D and Supplementary Figure 3B). We ascertained a mutually exclusive pattern of some somatic mutations among the 3 clusters, suggesting the importance of eSNV-based investigations of somatic mutations, as different mutations in the same gene may result in different prognostic outcomes. For instance, 4 of 6 eSNVs in *CTNNB1* were enriched in cluster 1 patients and showed mutually exclusive patterns. Notably, Liu *et al.* [8] reported that *CTNNB1* is a somatic driver that characterizes an aggressive subgroup in endometrioid-type endometrial cancer, which is enriched in cluster 1. However, a *CTNNB1* mutation on rs12191339 was observed only in cluster 2 patients, which correlated with a poorer survival rate. In addition, although most patients possessing *TP53* mutations were enriched in cluster 2, this confirms previous results that the *TP53* mutation is linked to poorer outcomes; however, the *TP53* rs121913343 variation was only observed in cluster 1 patients. Taken together, these results support the significance of assessing eSNV-based mutational profiles (compared to gene-based mutational profiles) in order to gain biological insights into disease profiles.

To identify cooperative dysregulation of eSNVs and gene transcription which may contribute to tumorigenesis and cancer progression, we derived a cluster-centric pairwise mutation-mutation correlation analysis based on β-coefficients of transcript levels of associated genes (Supplementary Figure 3C). As a result, eSNVs were categorized into 4 consensus clusters and we identified 3,706 significant pairwise co-occurrences (453 eSNVs), with overlapping correlated gene numbers ranging 10∼78 (Supplementary Figure 3D).

To clarify the SL interaction profile in endometrial cancer, we first indicated bimodal genes in the transcription profile, and then nominated candidate SL pairs according to the bimodal expression exclusively with an eSNV-based mutation. We identified 203 significant candidate SL eSNV-gene pairs, which contained 129 unique eSNVs (mapped to 109 genes) and 63 unique bimodal genes (Supplementary Table 4).

**Figure 5:**
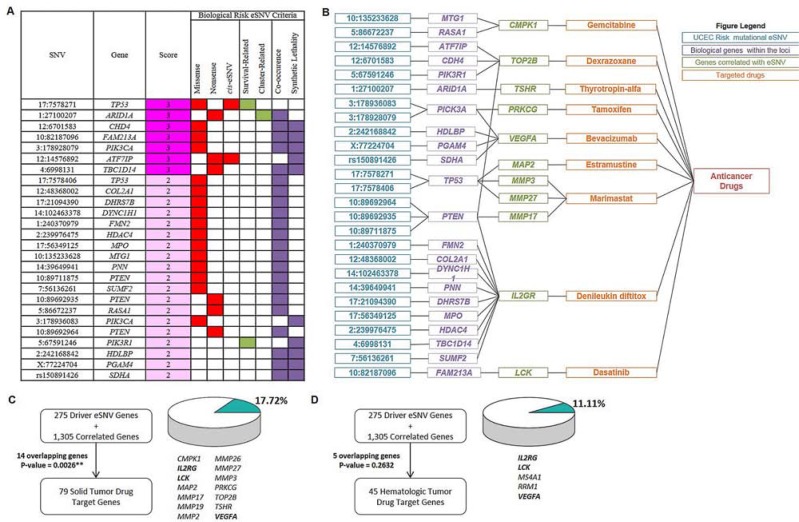
Biological candidate signatures for drug-repurposing to endometrial cancers **A.** Prioritized biological uterine corpus endometrial carcinoma (UCEC) candidate eSNVs (partial). eSNVs in E are listed, and summary scores derived from 7 criteria are shown. Filled boxes indicate fulfilled criteria. eSNVs with a score ≥ 2 were defined as “biological UCEC risk mutational eSNVs”. For complete information, see Supplementary Table 5. **B.** Connection plot showing relationships of identified eSNVs (blue), located genes (purple), and genes whose expression levels significantly correlated with the eSNV mutation status (green) with approved antineoplastic drugs (orange). eSNVs that were correlated with transcript levels of genes outside of *MMP* gene family are shown. For eSNVs that were solely correlated with transcript levels of the *MMP* gene family, see Supplementary Figure 4F. **C., D.** Figures showing numbers of overlapping genes between the query gene list constructed from 275 driver eSNV genes and 1,305 correlated genes and solid-tumor drug target genes (C) and hematologic-tumor drug target genes (D) Overlapping genes are listed, and proportions of numbers of overlapping genes to total drug target genes of solid tumors and hematologic tumors are visualized. *P* values were calculated by a hypergeometric test. **P < 0.01.

According to these findings, we adopted a scoring system based on the following 7 criteria to prioritize each of the 542 eSNVs (Figure 5A, Supplementary Figure 4A–4D and Supplementary Table 5): (i) missense eSNVs (*n* = 308); (ii) nonsense eSNVs (*n* = 74); (iii) *cis*-eSNVs (*n* = 3); (iv) eSNVs prioritized by correlation to survival (*n* = 13); (v) eSNVs prioritized by correlation to identified mutational clusters (*n* = 20); (vi) eSNVs prioritized by consensus clustering followed by a co-occurrence analysis (*n* = 453); and (vii) eSNVs prioritized by SL (*n* = 129). In total, 394 (72.69%) eSNVs had a score of ≥ 2, which mapped to 275 gene loci and correlated with transcript levels of 1,305 genes (Supplementary Figure 4E). We defined these eSNVs as biological UCEC risk mutational eSNVs.

Finally, we integrated drug target information [9–11] with UCEC biologically risky gene profiles constructed by 275 driver genes and 1305 associated genes. As anticipated, 14/79 (17.72%, hypergeometric test *p* = 0.0026) genes overlapped with drug target genes of approved solid-tumor drugs, including *CMPK1, IL2RG, LCK, MAP2, MMP17, MMP19, MMP2, MMP26, MMP27, MMP3, PRKCG, TOP2B, TSHR*, and *VEGFA*. These 14 genes were targets of 9 solid-tumor drugs (Figure 5B–5C and Figure S4F in Supplementary Figure 4F). These results indicated the rationality of clinical implementation of pharmacogenomic aspects into pharmacotherapy of these drugs by considering the heterogeneity of mutational profiles across endometrial cancer patients.

We also assessed how approved drugs for hematologic tumors, which were assigned as a control in our study, might be linked to biological UCEC risk mutational profiles. We highlighted 5 (11.11%, hypergeometric test *p* = 0.2632) genes from the UCEC profile that were drug target genes of approved hematologic-tumor drugs (45 genes in total, Figure 5D and Supplementary Figure 4G). Comparing overlapping genes between solid-tumor drug target genes and hematologic-tumor drug target genes revealed that 3 genes (*IL2RG, LCK*, and *VEGFA*) were duplicated, which accounted for 60% (3/5, hypergeometric test *p* = 0.0110, Supplementary Figure 4H) of overlapping genes from the UCEC profile connected to hematologic-tumor drug target genes. Taken together, these results provided biological plausibility for repositioning cancer drugs for other indications to endometrial cancer pharmacotherapy.

## DISCUSSION

In this study, first, we profiled UCEC-related driver somatic mutations in a personalized fashion, and gene transcript levels were associated with mutation statuses of identified eSNVs. Second, we showed that patients’ mutation profiles can be used to stratify UCECs, further linking disease aggressiveness and patient prognoses, thus providing a rationale to facilitate the fast-tracking of potential therapeutic targets in endometrial cancer. Third, we emphasized the importance of depicting somatic mutation profiles in an SNV-based manner, as different mutations in the same genes may link to distinct aggressiveness and prognostic outcomes in cancer patients. Finally, we adopted a scoring system to prioritize eSNVs, which may contribute to therapeutic switching.

In 3 identified endometrial cancer-related *cis*-eSNVs, we mentioned that alterations in *TP53* (*tumor protein p53*) are well-studied in endometrial cancer, and our results revealed that 14 eSNVs were significantly correlated with transcript levels of 267 genes, demonstrating a wide regulatory role of *TP53* somatic alterations in endometrial cancer. *ATF7IP* (*activating transcription factor 7 interacting protein*), a multifunctional nuclear protein associated with heterochromatin, was reported to be associated with testicular germ cell cancer [12]. Two eSNVs (chr12:14576892 and chr12:14649176) on *ATF7IP* were identified to cause dysregulation of 100 genes. Therefore, our study suggested *ATF7IP* as a new susceptibility locus in endometrial cancer. It is noteworthy that *TP53* and *ATF7IP* were annotated as transcription function-related genes (Figure 2E), which further explained thefunctional impacts of their alterations on downstream expression levels of many genes. In addition, 1 somatic alteration (chr8:21827087) on *XPO7* (Ran GTPase binding protein) was identified to be expression-associated in endometrial cancer, which was correlated with expression levels of 32 genes. Indeed, *XPO7* has been shown to be associated with serous epithelial ovarian cancer patient prognoses [13]. Here, we figured that the *TP53-, ATF7IP-*, and *XPO7*-associated expression profiles contribute to the discrepancy mutation statuses of these genes, demonstrating a wide range of perturbations of gene expression levels caused by somatic alterations.

In 13 identified cluster-related signatures, *TP53, CTNNB1, PIK3CA, ARID1A, FBXW7*, and *PPP2R1A* (which accounted for 65.0% (13/20) of eSNVs) are well-established recurrently mutated genes in endometrial cancers [5]. In this study. we demonstrated that these mutations embody the disease aggressiveness of endometrial cancers. Since late-stage (stages III + IV) and serous-like endometrial cancer patients were enriched in cluster 2 compared to clusters 1 and 3, the recurrent gain of these mutations may therefore be reflected by the disease progression, and further linked to disease aggressiveness in patients. To clarify the eSNVs that had the strongest prognostic values, we discovered 13 prognostic-related signatures (mapped to 10 genes) that could predict survival outcomes of endometrial cancer patients (see Supplementary note: *13 prognostic-related signatures*). Notably, when comparing the prognostic potentials between identified clusters and 13 prognostic-related signatures, we ascertained a higher clinical value of 13 prognostic-related eSNVs compared to mutational clusters, with summary measures of the AUC equal to 0.736 and 0.618, respectively. A time-dependent AUC also revealed that these 13 eSNVs possessed higher abilities to predict survival compared to mutation clusters, with first-year AUC values equal to 0.76 and 0.58, respectively. Taken together, we have enumerated 31 cluster-related and/or prognostic-related eSNV-based mutational signatures, and linking their mutational changes to various genes transcription level changes can highlight the underlying mechanism of how these somatic alterations incarnate disease aggressiveness and prognostic potential in endometrial cancer patients.

Most cancer-related mutational studies were conducted in a gene-based manner. Here, we adopted an SNV-based association test to gain more-detailed insights into endometrial cancer biology. After determining 31 cluster-related and prognosis-related signatures, we observed that most of the eSNVs showed clear mutual exclusivity between clusters, which correlated with different clinical outcomes (Figure 4D). As an illustration, somatic alterations, including chr4:153249510 (*FBXW7*), chr5:67591246 (*PIK3R1*), rs28934576 and rs11540652 (*TP53*), rs121913399 (*CTNNB1*), and chr19:52716323 (*PPP2R1A*), were specifically enriched in cluster 2, which was linked to higher tumor aggression and poor prognoses. However, rs121913343 in *TP53* and rs121913403, rs28931588, and 121913413 in *CTNNB1* were specifically enriched in cluster 1, which was identified to be less aggressive (see Supplementary note: *SNV-based somatic mutation profiles*).

We employed 7 biological criteria to prioritize eSNVs and construct biological risk candidates for drug discovery (Figure 5A–5C). Among 1,580 genes (275 driver genes and 1,305 correlated genes), 14 were determined to be solid-tumor drug target genes. In total, 9 drugs, including gemcitabine, dexrazoxane, thyrotropin-alfa, tamoxifen, bevacizumab, extramustine, marimastat, denileukin diftitox, and dasatinib, were identified to bind endometrial cancer-relevant altered genes that were associated with somatic mutations (Supplementary Table 6). We interpreted the findings from 2 following aspects. First, our result suggested a possibility for indication switching for endometrial cancer. For the solid-tumor drugs identified, most were approved therapeutic agents for other cancer types (e.g., breast cancer [tamoxifen], colorectal cancer [bevacizumab], and prostate cancer [estramustine]). For endorsement of re-profiling of our findings to endometrial cancer, a *VEGFA* inhibitor, bevacizumab, was shown to be well tolerated and active in recurrent or persistent endometrial cancer based on progression-free survival at 6 months [14]. A phase II randomized clinical trial is currently ongoing to assess the antitumor efficacy of combining bevacizumab with conventional chemotherapies in advanced or recurrent endometrial cancer (ClinicalTrials.gov identifier: NCT01770171). In addition, gemcitabine was shown to be well tolerated and showed modest activity in advanced endometrial cancer [15]. In addition, a retrospective phase II trial also revealed a high potency of combining gemcitabine with cisplatin in endometrial cancer patients [16]. Estramustine, an anti-microtubule chemotherapeutic agent was identified in our study, and was shown to exert growth-inhibitory effects in endoplasmic reticular-positive endometrial cancer cells *in vitro* [17]. Interestingly, tamoxifen was identified in our study, and it is widely used to treat breast cancer, and was shown to increase the endometrial cancer incidence. The results should be carefully interpreted as the mechanism of endometrium hyperplasia induction is due to the weak estrogenic effect of tamoxifen but not the target of tamoxifen, i.e., *PRKCG*, which was identified as a potential therapeutic biomarker in our study. In addition, low-grade endometrial cancer was also demonstrated to respond to tamoxifen treatment [18]. Here, we noted the pitfall of drug repurposing based solely on aberrant molecular signatures without considering multiple and complex biological effects of therapeutic agents. Nonetheless, we have described a powerful prioritization method for identifying promising therapeutic candidates which may benefit endometrial cancer treatment.

Second, we mentioned a latent pharmacogenomic issue for the identified therapeutic agents when implementing clinical cancer treatment. Investigation of somatic alterations revealed a highly heterogeneous cancer profile across patients, providing a rationale to incorporate somatic mutation information into clinical use for more-efficient cancer treatments. For example, we discovered that *LCK* is a candidate therapeutic gene in endometrial cancer, and the expression level of *LCK* was significantly correlated with a somatic alteration of chr10:82187096. *LCK* is inhibited by dasatinib, a Src family protein (including p56^Lck^) and an EphA2 receptor blocker that inhibits a large array of targets. In support of dasatinib's therapeutic switching, a clinical trial (ClinicalTrials.gov identifier: NCT01440998) of dasatinib in late-stage and recurrent endometrial cancer is ongoing [2]. Due to the promising molecular scheme, the clinical applicability of dasatinib in cancer treatment may therefore depend on harbored mutations of patients.

There have some limitations of this study. Based on its retrospective design, we showed the associations among identified drivers and clinical variables, further functional studies (in basic field or clinical study) and validations are required to assess the possibility of clinical application and implementation of our findings.

In summary, our study provides new insights into pharmacogenomic-guided pharmacotherapies based on driver eSNV profiles of endometrial cancer patients and closes the gap between oncogenomic research and traditional personalized trials.

## CONCLUSIONS

In summary, our results reveal an integrative framework of correlation between SNV-based somatic mutations and transcripts’ levels in endometrial cancer. Since a number of driver eSNVs showed different aberration potential to transcripts, we illustrated the correlation between driver eSNV and clinicopathological features, e.g. disease stage, tumor histology and patients’ survival. In addition, we prioritized driver eSNVs and together their correlated probes, showing that these genes were enriched in known targets of approved solid tumor drugs. This approach can narrow down the candidate drugs before conducting clinical trial, thereby providing new avenues for the drug development of endometrial cancer, boosting drug discovery process and providing potential gene targets and drug candidates for the drug-repurposing and treatment of endometrial cancer. Besides, this approach can also be applied to other cancer types to prioritize drug candidates and facilitate their clinical applications and implementations.

## MATERIALS AND METHODS

### Datasets

Our study included 219 UCEC patients with primary tumor RNA expression data and mutation data (Supplementary Figure 1A). We queried the RNA-sequencing expression profiles (assay platform: RNASeq vers. 2) from TCGA public database with *TCGA-Assembler* package [19] Normalized relative square error of the mean (RSEM) gene expression data of primary solid tumors (370 samples) and normal adjacent solid tissues (11 samples) were queried and further processed by the “ProcessRNASeqData” function implemented in *TCGA-Assembler* package. A principal component analysis was conducted with the *ade4* package to avoid artifactual results caused by sample relatedness, and the top 3 principal components using 370 UCEC patients’ primary tumor part expression values were illustrated by a 2-way scatter plot (Supplementary Figure 1B). Methylation profiles (Infinium HumanMethylation450 BeadChip) and related annotation files (https://tcga-data.nci.nih.gov/tcga/tcgaPlatformDesign.jsp) of UCEC patients, including 256 samples of primary solid tumors and 26 normal adjacent solid tissues, were download form TCGA data portal (https://tcga-data.nci.nih.gov/tcga/) in data level 3 [5]. Methylation data were further processed with *TCGA-Assembler*. In brief, measurement of a CpG site was duplicated if it corresponded to more than 1 gene using the “ProcessMethylation450Data” function, and then gene-based average methylation values were calculated based on the beta value within 1, 500 bp of a transcription start site using the “CalculateSingleValueMethylationData” function. As a result, average beta values of 15, 625 genes were obtained for further study. TCGA UCEC somatic copy number alteration (SCNA) data of 492 samples generated by the GISTIC algorithm and mutation profiles of 248 TCGA endometrial cancer patients, represented as SNVs were also queried from the public access TCGA database (https://www.synapse.org/#!Home:0) and further processed in R. The genomic coordinate was based on NCBI build 37 (hg19). The expression or methylation value of primary tissue was then normalized by the expression or methylation value of adjacent normal tissue to construct a standardized absolute differential expression or methylation profile [20]. Furthermore, sole SNVs, SNVs annotated as insertion/deletion or germline/others, and all SNVs located outside of chromosomes 1∼23 were excluded from the mutation profile. Then, we removed genes located within the major histocompatibility complex (MHC) region in the expression, SCNA, methylation, and mutation profiles. After data sanitization, UCEC samples with were retrieved for further eSNV identification. A density plot of methylation, somatic copy number alteration, and expression profiles were constructed based on the probability density for evaluation of the data distribution (Supplementary Figure 1B). Besides, we conducted principal component analysis (PCA) to assess the underlying sample substructure (Supplementary Figure 1C).

### SNV-based correlation analysis

Correlations of somatic mutations and RNA expressions in UCEC patients were carried out to reveal the disease biology which contributed to drug discovery. We selected 219 samples with both mutation and expression data from TCGA UCEC dataset. The following analyses were all conducted using these 219 sample profiles if not specified. In order to adjust for other genetic determinants that also correlate with transcript abundances, we further extracted 128 samples with fully annotated somatic copy number alteration and methylation profiles to undergo a multivariate linear regression analysis (Figure 1A).

We conducted a 2-step linear regression analysis modified from Li et al. [21] to examine the correlation between somatic SNVs and gene expressions in endometrial cancer patients. For each gene *i*, a multivariate linear regression (Equation 1) was first applied to compute the residual expression value (ε_*i*_) of somatic copy number alterations (*SCNA_i_*) and relative methylation levels (*M_i_*). Then, residual expression values (ε_*i*_) were regressed by each mutation locus *j* (Equation 2) to identify expression-associated (e)SNVs, as shown in the following equations:

$${\text{RT}}_{i}={\text{SCNA}}_{i}+M_{i}+\varepsilon_{i}\text{ and}$$ (1)

$$\varepsilon_{i}={\text{SNV}}_{j+}{ɣ}_{j};$$ (2)

where *RT_i_* is the relative transcript abundance, *SCNA_i_* is the somatic copy number alteration GISTIC score, *M_i_* is the relative methylation beta value, ε_*i*_ is the residual relative expression value, *SNV_j_* is a single-nucleotide variation, and ɣ_*j*_ is the random error.

In Equation 1, an analysis of variance (ANOVA) test was used to separately estimate the effects of somatic copy number changes and methylation on the transcript level, and therefore the mean of explanation of total variation of both genetic determinants was calculated. In addition, Benjamini & Hochberg adjusted *p* values were used to control the false-positive rate. In Equation 2, we tested for 78, 571, 548 pairs of SNV-gene associations by adopting a linear regression model (5, 824 SNV and 13, 491 genes), followed by the Bonferroni correction for multiple testing corrections. A genomic inflation factor lambda value was calculated (carried out with the *snpStats* package) to explore the possibility of cryptic sample relatedness that may bias the results of the association study. We then categorized significant associations based on their eSNV-gene relationships. We defined SNVs located within the gene that showed an association with the transcript level as “*cis*-acting eSNVs” and SNVs located outside of the gene showing correlations with the expression value as “*trans*-acting eSNVs”. We also intersected significant genes correlated with 3 genetic determinants (CpG methylation, somatic copy number alterations, and mutational eSNVs). For data presentation, pie charts were constructed with the *plotrix* package and a Venn diagram was illustrated with Venny (http://bioinfogp.cnb.csic.es/tools/venny/).

### Gene-based prioritization

After performing an SNV-based association analysis to identify expression-associated somatic alterations, we used the package *DawnRank* [20] to identify candidate personalized driver mutational genes in 219 TCGA UCEC samples (with both expression and mutation profiles available). RNA-sequencing expression data of primary tumors were normalized by available adjacent normal solid-tissue data using the “DawnNormalize” function, which returns a standardized differential expression matrix. The full gene network required by the *DawnRank* algorithm was downloaded from the *DawnRank* homepage (http://bioen-compbio.bioen.illinois.edu/DawnRank/) which contained curated information from Reactome Database (http://www.reactome.org/), the Pathway Interaction Database (http://pid.nci.nih.gov/), and Kyoto Encyclopedia of Genes and Genomes (KEGG) database (http://www.genome.jp/kegg/). In short, the *DawnRank* algorithm evaluated the connectivity and number of differentially expressed genes to measure the impact of a mutation, utilized a dynamic damping factor to rank mutated genes based on their ability to perturb downstream genes, and returned personalized ranked mutated gene lists of every single UCEC sample. After the *DawnRank* analysis, we adopted the *maxstats* package to calculate maximally selected rank statistics of each patient in order to distinguish passenger from driver mutations with a cutoff of 0.95. In addition, predefined genes identified by TCGA, including *TP53, CTNNB1, PIK3CA, PIK3R1, PTEN, POLE, ARID1A, KRAS, CTCF, FBXW7, PPP2R1A*, and *ARID5B* (*RPL22* was excluded as no SNV was detected to be correlated with its transcript level), were collaboratively included to prioritize eSNVs based on their corresponding gene loci. The number of candidate personalized driver genes identified by *DawnRank* was further correlated with UCEC patients’ clinical profiles (disease stage, tumor histology, and tumor grade) by a Kruskal-Wallis rank sum test, and pairwise significance was determined by a Wilcoxon test (with the Bonferroni correction method for multiple testing corrections).

We then conducted an over-representation analysis (ORA) based on gene ontology (GO) biological process (BP) terms using the *ClusterProfiler* package [22]. We selected 0.01 as the significant Benjamini & Hochberg adjusted *p* value cutoff to identify significantly enriched GO terms. We also downloaded a gene list of 1, 470 transcription factors (TFs), 297 transcription co-factors, and 118 chromatin-remodeling factors from The Animal Transcription Factor Database (*Homo sapiens*, AnimalTFDB) on 4 May 2015 [23] a comprehensive transcription factor database. We categorized 542 eSNVs into 2 categories: 99 TF-related eSNVs which mapped genes were annotated to be essential in gene transcription processes or gene transcription regulation (i.e. genes belonging to one of either “TF”, “transcription co-factor”, or “chromatin remodeling factor”) and remaining 443 non TF-related eSNVs. The proportions of mutation types (missense, silent, nonsense, 3′ untranslated region (UTR), intron, RNA, splice site and 5′ UTR mutations) in all, TF-related, and non-TF-related categories were separately calculated.

### Mutation cluster analysis

We conducted a clustering analysis based on 542 prioritized driver eSNVs to categorize UCEC patients into 3 groups based on their mutation profiles. In considering the sparse nature of the mutation matrix, we applied an unsupervised spherical k-means algorithm as implemented in the *skmeans* package to the mutation data with parameters *k* = 3 and method = ‘pclust’. The clustering result was visualized with the *NMF* package, with UCEC samples seriated within each cluster with the *seriation* package. A heatmap was constructed based on identified patient clusters and 542 eSNVs. In the heatmap visualization, black indicates mutations that occurred, while white indicates that no mutation was recorded. Unsupervised agglomerative hierarchical clustering of eSNVs was conducted with the parameters scale = ‘row’, distfun = ‘correlation’, and hclustfun = ‘ward.D’. We then correlated the 3-cluster configuration with patients’ clinical profiles including the disease stage, tumor histology, tumor grade (by Fisher's exact test), and survival data (by a Cox-regression test). Cox-proportional hazard models in the *rms* and *survival* packages were applied to evaluate the prognostic potential of the identified cluster against progression-free survival data with or without adjusting for the disease stage and tumor histology. The Kaplan-Meier estimator was illustrated with the *ggplot2* package. To further elucidate the prognostic potential of the identified cluster, we carried out a 10-year time-dependent receiver operating characteristic (ROC) curve analysis, with time-dependent accuracy summarized through correct classification rates defined as sensitivity and specificity was calculated, and calculated the cumulative case/dynamic control area under the ROC curve (AUC) as implemented in the *survivalROC* package [24]. ROC curves constructed using the true positive rate (y-axis) and false positive rate (x-axis) can be utilized to evaluate the performance of a predictor against censored survival data. C-statistics which had scores of 0.5∼1.0 were calculated to assess the accuracy of the identified clusters against the survival phenotype. The ROC curve lay on the 45° line indicated that the analyzed classifier was unable to predict survival outcomes. In addition, the summary measure of the censoring-adjusted C-statistic was calculated by Uno's method as implemented in the *survAUC* package.

### Prioritization of biological candidate eSNVs

To identify cluster-related eSNV signatures, we applied Fisher's exact test to correlate 542 eSNVs with identified patient clusters and selected a significance threshold of 0.05. Additionally, prognostic-related signatures were identified by applying a Cox-proportional hazards model. The null hypothesis, that there was no correlation between the mutation status and survival, was tested. We selected eSNVs that showed significance (with a Cox-regression *p* value of < 0.05) against progression-free survival with or without adjusting for clinical profiles. According to the 13 identified prognostic-related eSNV signatures, we classified patients into 2 groups: “with mutation(s)” (which mutation(s) could be found in at least one of the prognostic-related signatures) and the remaining as “without a mutation”. To construct the Kaplan-Meier distribution, UCEC patients were assigned to 2 groups, and the significance level of the progression-free survival difference between the 2 groups was calculated based on the Cox-regression model. Furthermore, to assess how well the 2-group configuration predicted the survival time for UCEC patients, a 10-year cumulative/dynamic ROC analysis of prognostic-related eSNV signatures was carried out, and summary measures of AUC were also calculated as described above.

To access the cooperative dysregulation of somatic mutations and relative expressions, we carried out a pairwise eSNV-eSNV correlation analysis based on the overlapping number of their associated genes. Fisher's exact test on a 2-by-2 contingency table was used to identify significant mutational eSNV pairs that co-occurred (Bonferroni corrected *p* < 0.05). Then, we applied 2 additional analytical steps to minimize latent false positive coincident eSNV pairs. In considering the direction and magnitude of the coefficient, an unsupervised consensus clustering analysis of eSNVs using associated genes (beta coefficients) was conducted. We used the *ConsensusClusterPlus* package with 80% subsampling over 1, 000 iterations of k-means upon Euclidean distance matrix for subclasses identification with parameter maxK = 7. We successfully identified 4 robust clusters that accounted for positive or negative relationships between the eSNVs and associated genes. We thus excluded eSNV pairs that belonged to different consensus clusters. We further excluded eSNV pairs with overlapping associated gene numbers of < 10 to declare significant co-occurrence.

We applied the *BISEP-BEEM* (bimodality subsetting expression-bimodal expression exclusive with mutation) algorithm as implemented in the *BiSEp* package to identify candidate synthetic lethality (SL). Two genes were defined as having SL if mutations in both genes caused cell death but a mutation in either gene alone did not lead to cell death. To harmonize the RNA-sequencing data to the downstream analysis, we used the *sRAP* package to normalize RPKM values of primary tumors in UCEC patients. We rounded the expression value to < 0.1 to avoid bias caused by low-coverage genes, followed by log_2_ transformation. The log_2_-normalized expression profiles of samples were inputted into *BiSEp* to detect bimodality (2 mixture components) and non-normality in all genes by model-based hierarchical clustering. Then, the BEEM algorithm was applied to detect eSNVs enriched in either of the bimodal gene expression modes. A threshold of Fisher's exact *p* value of < 0.05 was used to select candidate SL eSNV-gene pairs.

Each driver eSNV identified from UCEC patients was scored by the following 7 criteria (the adopted scoring system was modified from Yukinori Okada *et al.* [25]): (i) eSNVs were annotated as missense variants; (ii) eSNVs were annotated as nonsense variants; (iii) eSNVs showed significant associations with gene expressions that were located within associated genes (*cis*-acting); (iv) eSNVs were prioritized by identifying mutational clusters (cluster-related) with *p* < 0.05; (v) eSNVs were prioritized by a Cox-regression with or without adjusting for clinical profiles (prognostic-related) with *p* < 0.05; (vi) eSNVs were prioritized by a pairwise mutational co-occurrence analysis with adjusted *p* < 0.05 and which showed consensus associated gene profiles; and (vii) eSNVs were prioritized by a candidate SL eSNV-gene pair nomination analysis with *p* < 0.05. Correlations among these 7 criteria were calculated and visualized with the *corrplot* package, with a hierarchical clustering order for the correlation matrix. We carried out a pairwise significance test using a Phi correlation coefficient for the association among the 7 criteria (significant *p* < 0.01). We calculated the number of criteria that were satisfied and assigned a score to each driver eSNV, which ranged 0∼7. We defined those eSNVs with a score of ≥ 2 as “biological UCEC risk mutational eSNVs”.

### Candidate tumor drug mining

Drug target gene information was obtained from the DrugBank database (http://www.drugbank.ca/) and Therapeutic Target Database (http://bidd.nus.edu.sg/group/cjttd/) on 28 April 2015 [10, 26]. Drug target genes were filtered by several criteria including human species and targeted by approved drugs. We manually extracted genes which were annotated as the target of an antineoplastic drug based on the indication. Then, the drug target profile was separated into a “solid-tumor drug target profile” (*n* = 79) and a “hematologic-tumor drug target profile” (*n* = 45) based on annotated indications. The hematologic-tumor drug target profile was constructed based on the keywords ‘leukemia’, ‘lymphatic leukemia’, ‘multiple myeloma’, ‘lymphoma’, ‘leukemias and lymphomas’, and ‘hematologic malignancies’, while the remaining were extracted to build a solid-tumor drug target profile. We evaluated whether genes from the biologically risky gene profiles were pharmacologically therapeutic targets of approved solid-cancer drugs. In total, 275 genes (mapped by 394 prioritized biological eSNVs) and 1, 305 correlated genes were used to separately query corresponding drugs in the solid-tumor and hematologic-tumor drug target profiles. Overlapping genes between the query gene list and solid-tumor or hematologic-tumor drug target profiles were extracted and counted. In addition, drugs that targeted genes in the query gene list were also illustrated. Enrichment of overlapping genes extracted from the solid-tumor and hematologic-tumor profiles were calculated by a hypergeometric test.

### Statistical analyses

We performed all analytic workflows in this study under the R environment (http://www.r-project.org/ and http://cran.r-project.org/) and Bioconductor (http://www.bioconductor.org/).

## SUPPLEMENTARY MATERIALS FIGURES AND TABLES















## List of abbreviations

UCEC: uterine corpus endometrial carcinoma
TCGA: The Cancer Genome Atlas
eSNVs: expression- associated single nucleotide variations
RSEM: relative square error of the mean
SCNA: somatic copy number alteration
MHC: major histocompatibility complex
ANOVA: analysis of variance
KEGG: Kyoto Encyclopedia of Genes and Genomes
ORA: over-representation analysis
GO: gene ontology
BP: biological process
TFs: transcription factors
AnimalTFDB: The Animal Transcription Factor Database
UTR: untranslated region
ROC: receiver operating characteristic
AUC: area under the ROC curve
SL: synthetic lethality.
