# Supplementary material for: Integrative bioinformatic analyses of an oncogenomic profile reveal the biology of endometrial cancer and guide drug discovery
Source: Oncotarget. 2015 Dec 22;7(5):5909–23. doi: 10.18632/oncotarget.6716 (PMC4868730; doi:10.18632/oncotarget.6716)
Supplement: Supplementary file 1 [file oncotarget-07-5909-s001.pdf]

Integrative bioinformatic analyses of an oncogenomic profile reveal the biology of endometrial cancer and guide drug discovery

Supplementary Materials

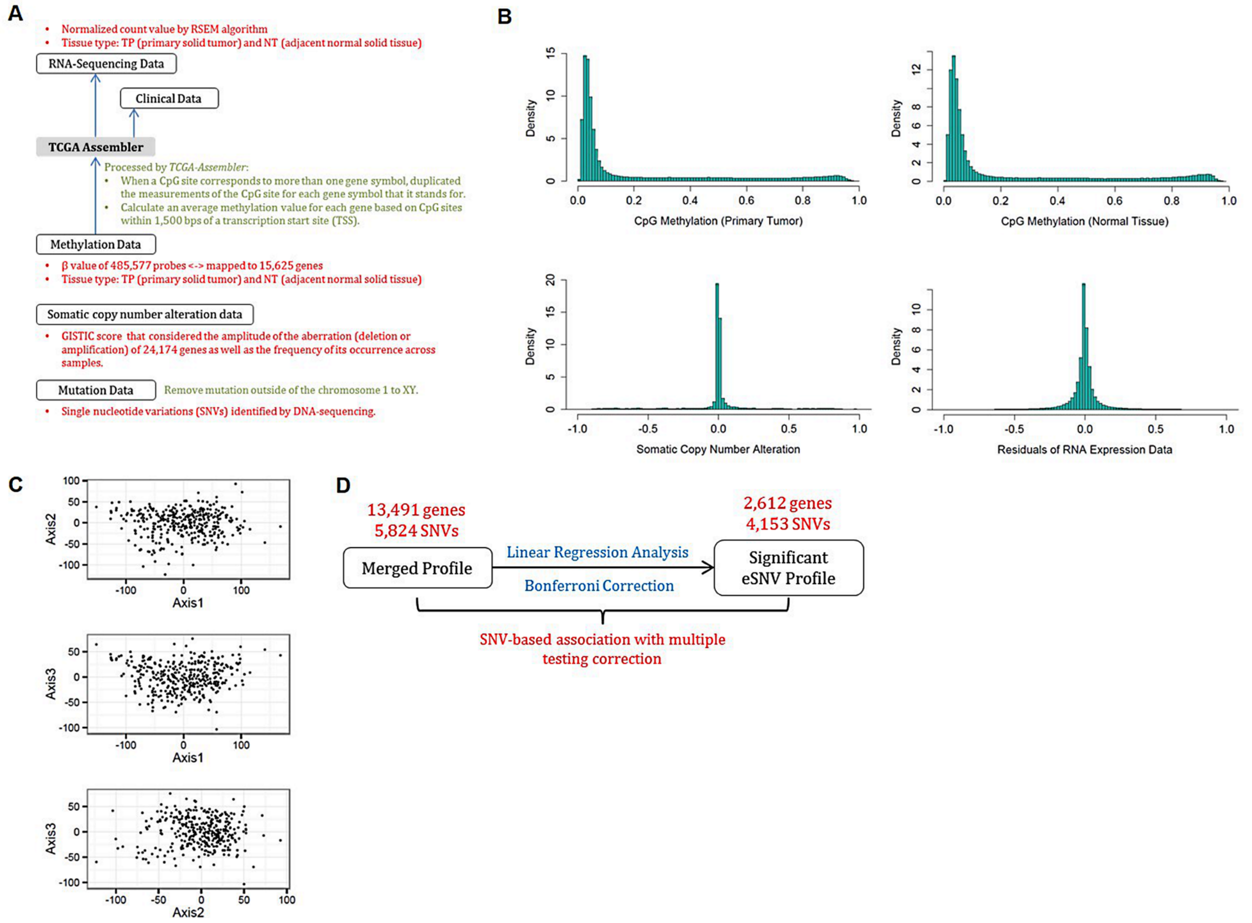

Supplementary Figure S1: Endometrial Cancer Data Sets. (**A**) Schematic showing data sanitization procedures for TCGA endometrial cancer data sets. Transcription sequencing data of primary tumours and normal adjacent tissues were queried and processed by *TCGA-Assembler*. UCEC patients' clinical data were also downloaded by *TCGA-Assembler*. Methylation (including available primary tumor tissues and tumor adjacent normal tissues), somatic copy number alteration (SCNA) and mutation profiles were downloaded from public TCGA database. Downloaded methylation data in data level 3 was further processed by *TCGA-Assembler* to calculate gene-based methylation value. (**B**) Principal component analysis (PCA) of 370 available expression profiles of primary tumor tissues. The first three principal components were used to assess the sample relatedness and underlying confounding factors. Based on the visualized plots, transcription profiles of endometrial cancer patients were well-clustered with no significant outlier was detected. (**C**) Density plots for measuring the distribution of SCNAs [bottom left], CpG methylation and residual expression data [bottom right]. CpG methylation profiles of primary tumor tissues and tumor adjacent normal tissues were illustrated separately [top left and top right]. Importantly, the distribution of residuals approximated normal distribution, and met the prerequisite for linear regression analysis to identify expression-associated single nucleotide variations. (**D**) Schematic showed the mutation analysis procedure. To identify significant expression-associated single nucleotide variations (eSNVs), linear regression analysis was conducted followed by Bonferroni correction. The association tests for mutation profile were SNV-based instead of gene-based. We tested the association between 5,824 somatic mutations to relative transcript level of 13,491 genes (phenotypes). As a result, 4,153 somatic mutations were identified to be expression-associated single nucleotide variant. The mutation status of these eSNVs was correlated to the expression level of 2,612 genes.

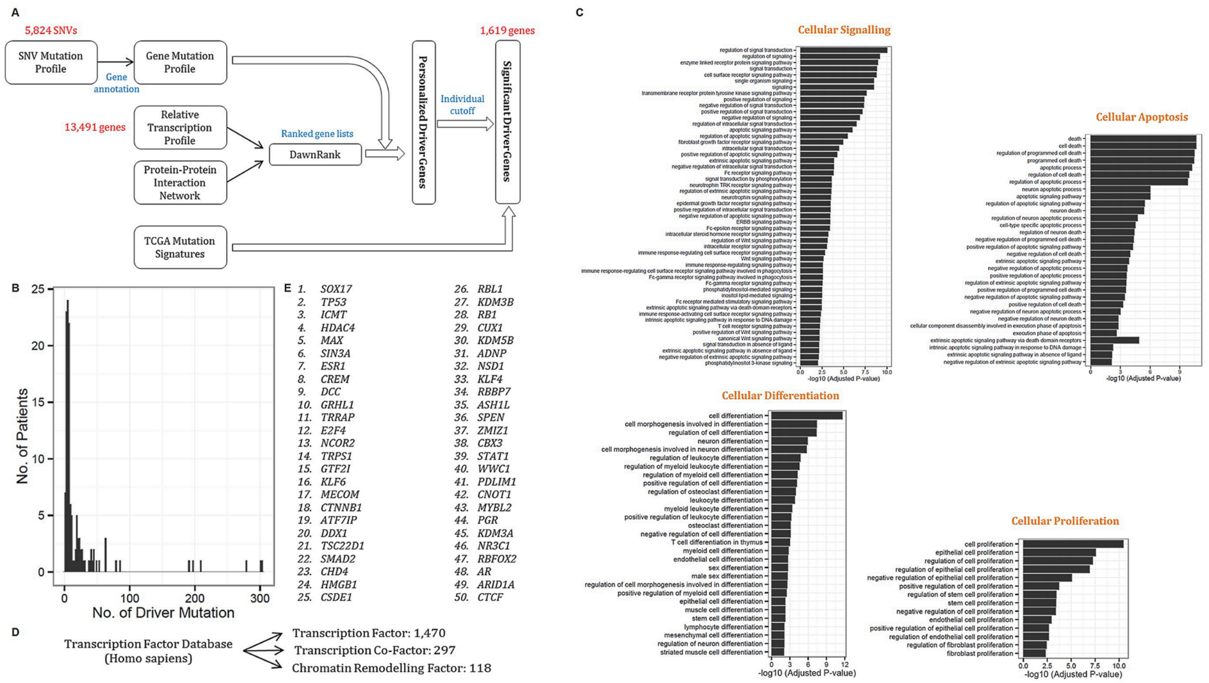

Supplementary Figure S2: Gene-based prioritization and mutation clusters identification. (**A**) Schematic showing gene-based prioritization procedures. *DawnRank* algorithm was applied for identifying candidate driver genes based on protein-protein interaction information (See *Materials and Methods*). By using *DawnRank* algorithm, personalized driver mutations were identified and we further applied individual cut-off to select significant personalized drivers. Besides, we also included twelve TCGA mutational signatures to aid in prioritization. (**B**) Bar plot showed distribution of number of driver mutations identified by *DawnRank*. As shown, a large proportion of patients contained less than 100 drivers, while a small amount of patients carried large amount of driver mutations (200 to 300). (**C**) Bar plots showing significantly over-represented Gene Ontology terms related to cellular signaling [top left], cellular apoptosis [top right], cellular differentiation [bottom left] and cellular proliferation [bottom right] biological processes. (**D**) Complete profile of AnimalTFDB (*Homo sapiens*), which including 1,470 transcription factors, 297 transcription co-factors and 118 chromatin remodelling factors. (**E**) 50 genes identified from AnimalTFDB. The eSNVs were first mapped to genes based on located loci, and then intersected with the AnimalTFDB *Homo sapiens* profile to identify genes with transcription-related function.

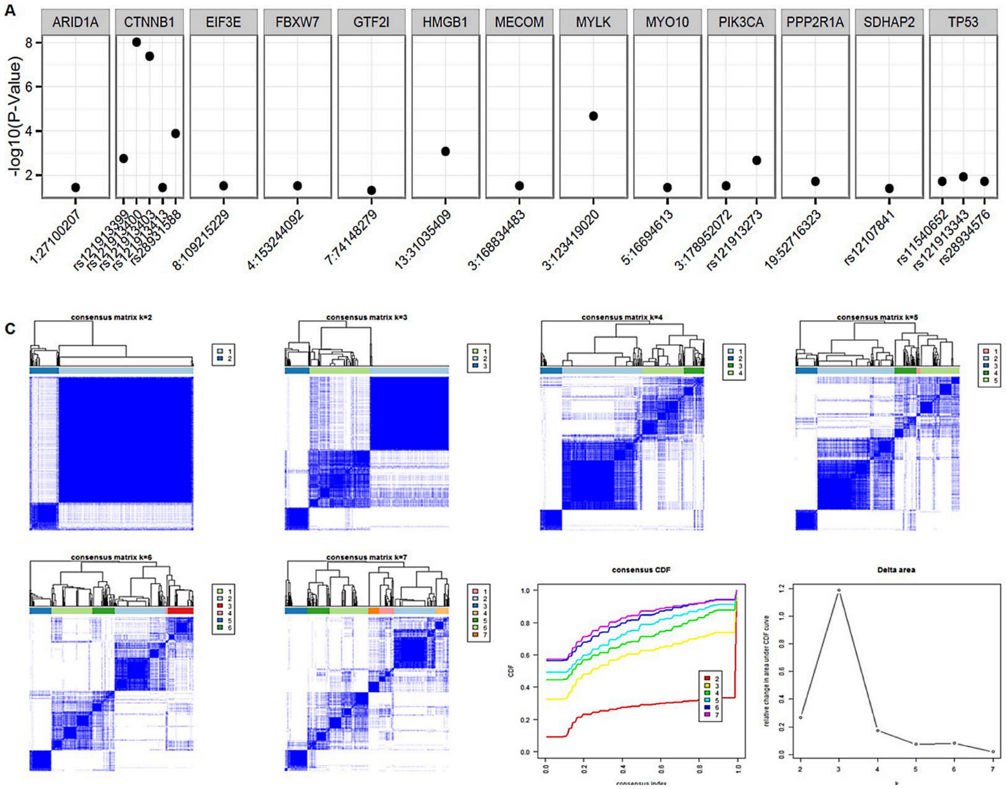

B

SNV	Gene	Cluser-Related	Prognostic-Related
1:27100207	*ARID1A*	•	
12:6701638	*CHD4*		•
rs121913399	*CTNNB1*	•	
rs121913400	*CTNNB1*	•	
rs121913403	*CTNNB1*	•	
rs121913413	*CTNNB1*	•	
rs28931588	*CTNNB1*	•	
8:109215229	*EIF3E*	•	
4:153249384	*FBXW7*		•
4:153249510	*FBXW7*		•
4:153244092	*FBXW7*	•	
7:74148279	*GTF2I*	•	•
13:31035409	*HMGB1*	•	
3:168834483	*MECOM*	•	
19:42853772	*MEGF8*		•
22:31322760	*MORC2*		•
3:123419020	*MYLK*	•	
5:16694613	*MYO10*	•	
10:50966551	*OGDHL*		•
rs121913273	*PIK3CA*	•	
3:178952072	*PIK3CA*	•	
5:67591246	*PIK3R1*		•
19:52716323	*PPP2R1A*	•	
10:89692802	*PTEN*		•
rs12107841	*SDHAP2*	•	
rs11540652	*TP53*	•	
rs121913343	*TP53*	•	
rs28934576	*TP53*	•	•
17:7578271	*TP53*		•
rs2893874	*TP53*		•
3:183524831	*YEATS2*		•

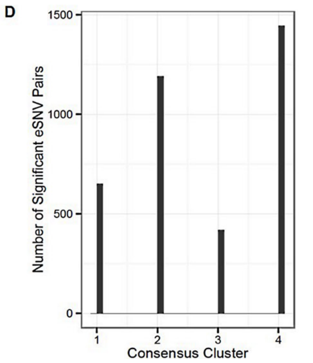

Supplementary Figure S3: Integrative analyses. (**A**) Scatter plots showed the significance of 20 mutational cluster-related signatures. The x-axis was somatic mutation and the y-axis was –log^{10} value of *p*-value. SNVs were plotted individually based on their located genes. (**B**) Table indicated 31 eSNVs that were either cluster-related or prognostic-related. Mutations including 7:74148279 (*GTF2I*) and rs28934576 (*TP53*) showed correlation to both the identified mutational cluster and progression-free survival. (**C**) Consensus clustering of eSNVs based on their correlated transcripts for co-occurrence analysis. The matrix contained beta coefficients of eSNVs against genes was used for identifying eSNVs that showed similar cooperative dysregulation pattern by correlated with the expression level of the same genes. The heatmaps of the consensus matrix for k equal to 2, 3, 4, 5, 6 and 7 were showed in following manner: consensus values ranged from zero (white) to one (blue), which represented never clustered together and always clustered together, respectively. Besides, consensus cumulative distribution function (CDF) plot and Delta area plot were also constructed to aid determination of number of consensus cluster (k). We chose k = 4 as there is no appreciable relative increase in consensus after k = 4. (**D**) Summaries of co-occurrence results. After determine the number of consensus cluster (k = 4), the number of significant eSNV pairs belong to each cluster was calculated.

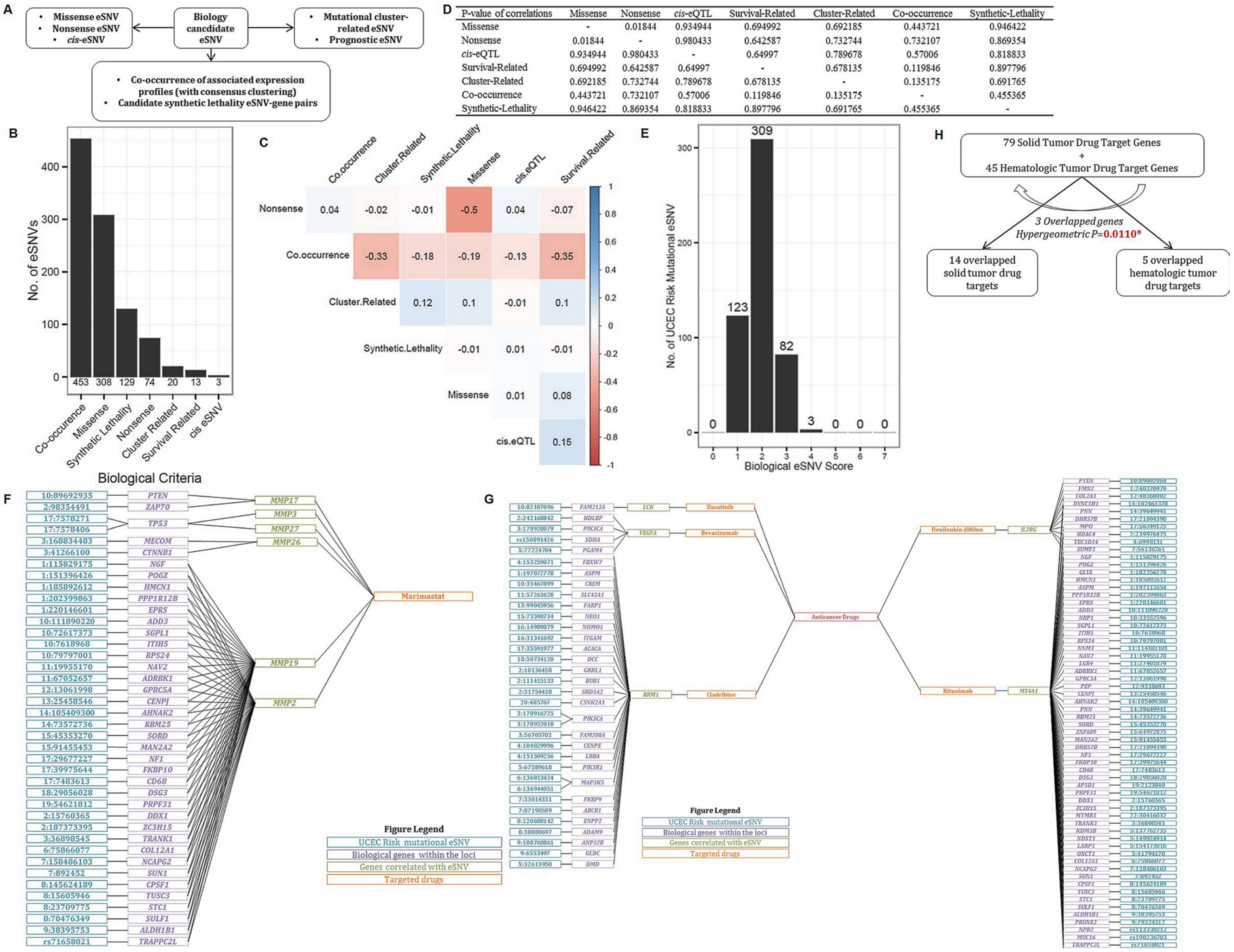

P-value of correlations	Missense	Nonsense	*cis*-eQTL	Survival-Related	Cluster-Related	Co-occurrence	Synthetic-Lethality
Missense	-	0.01844	0.934944	0.694992	0.692185	0.443721	0.946422
Nonsense	0.01844	-	0.980433	0.642587	0.732744	0.732107	0.869354
cis-eQTL	0.934944	0.980433	-	0.64997	0.789678	0.57006	0.818833
Survival-Related	0.694992	0.642587	0.64997	-	0.678135	0.119846	0.897796
Cluster-Related	0.692185	0.732744	0.789678	0.678135	-	0.135175	0.691765
Co-occurrence	0.443721	0.732107	0.57006	0.119846	0.135175	-	0.455365
Synthetic-Lethality	0.946422	0.869354	0.818833	0.897796	0.691765	0.455365	-

Supplementary Figure 4: Endometrial cancer drug re-purposing or discovery. (**A**) Schematic showing 7 criteria for biological candidate mutation prioritization (SNV-based) in endometrial cancer patients. (**B**) Bar plot showed the prioritization criteria of biological risk mutational eSNVs. (**C**) Correlogram indicated the pairwise Phi correlation coefficient between seven criteria. The blue colour denoted a positive correlation while red colour denoted a negative correlation. (**D**) Table showed the *p*-value of correlation between seven criteria to evaluate the pairwise dependency across 7 criteria. (**E**) Histogram distribution of biological eSNV score. (**F**) Connection plot showing relationship between identified eSNVs (blue), located genes (purple), genes that expression level significantly correlated with eSNV mutation status (green) and approved antineoplastic drugs (orange). The eSNVs that correlated to the transcript level of *MMP* gene family were showed (solid tumor drug targets). (**G**) Connection plot showing relationship between identified eSNVs (blue), located genes (purple), genes that expression level significantly correlated with eSNV mutation status (green) and approved haematological antineoplastic drugs (orange). (**H**) Overlap of antineoplastic drug target genes with targets of overlapped solid tumor drugs and targets of overlapped hematologic tumor drugs. *P*-value was calculated by hypergeometric test. **P*-value < 0.05.

Supplementary Table S1: Results of linear regression analysis between somatic alterations and genes' expression level.

Supplementary Table S2: Over-representation evidence of enriched Gene Ontology (GO) terms of 542 eSNVs and 1894 associated genes in biological processes (BP).

Supplementary Table S3: Identification of prognostic-related signatures.

Supplementary Table S4: Candidate synthetic lethal eSNV-gene pairs.

Supplementary Table S5: Score of prioritized biological candidate risk eSNVs (score ≥ 2) calculated by 7 criteria.

Supplementary Table S6: Identified solid tumor drug target genes and corresponding therapeutic agents based on 275 driver eSNV genes and 1305 correlated genes.

SUPPLEMENTARY NOTE

13 prognostic-related signatures

In 13 identified prognostic-related signatures, three (chr17:7578271, rs28934576, and rs28934874) were located in *TP53*. Two of the prognostic-related eSNVs (chr4:153249384 and chr4:153249510) were in *FBXW7*. *FBXW7* is a tumor suppressor that mediates ubiquitin-dependent proteolysis of cyclin E1, c-Myc, c-Jun, and Notch in endometrial cancer [1]. In *CHD4*, a gene plays an important role in epigenetic transcriptional repression, somatic alteration on chr12:6701638 were detected to be correlated with survival. In addition, we also found prognostic-related somatic alterations on *PIK3R1* and *PTEN*, which are well-known cancer drivers in endometrial cancer [2]. Also, mutations on *YEATS2*, an epigenetic regulator were identified to be prognosis-related. Other identified prognosis-related signatures were located in genes that are less characterized in endometrial cancer, including *OGDHL, MEGF8, MORC2*, and *GTF2I. OGDHL* is an AKT-dependent nuclear factor (*NF*)-*κB* signaling modifier, and was suggested to act as an anti-proliferative gene [3]. *MEGF8* is a membrane protein gene that participates in developmental regulation and cellular communication [4]. *MORC2* is mainly located in nuclei, it was reported to act as a transcriptional repressor in cancer cells, and its signaling integrates extracellular signals and nuclear processes during DNA damage [5, 6]. Lastly, the *GTF2I* mutation was reported to occur at high frequencies in thymic epithelial tumors [7], suggesting an oncogenic role of *GTF2I* in cancer.

SNV-based somatic mutation profiles

We conducted several analyses to link gene expression levels to somatic alterations in an SNV-based manner to further consummate the explanation of mutational impacts on cancer transcriptomic profiles. First, we conducted an SNV-based linear regression test and revealed that different eSNVs in a gene may exert different effects (quantified by the β-coefficient) on the same genes' transcript levels. We use *TP53* as an example: chr17:7577563 and rs121913343 were both significantly correlated with transcript levels of *LAG3* and *SLAMF7*, but the effects differed (β-coefficient = 0.32 vs. 0.17 for *LAG3* and 0.46 vs. 0.24 for *SLAMF7*, respectively). In addition, chr17:7578271 and chr17:7578406 also showed different effects on *NLRP7* (β-coefficients = 0.50 and 0.35, respectively). Second, we classified eSNVs by consensus clustering on β-coefficients to genes and discovered that eSNVs on the same genes may be grouped into different consensus clusters based on their correlated transcript profiles. Based on these findings, we finally conducted SL pair identification by bioinformatics means in an eSNV-based fashion. We found that 129 eSNVs had potential SL to other genes. Although further validation is needed, these findings consolidate our contention that cancer-relevant somatic mutations which have SL to genes could be SNV-specific.

REFERENCES

1. Akhoondi, S., D. Sun, N. von der Lehr, S. Apostolidou, K. Klotz, A. Maljukova, D. Cepeda, H. Fiegl, D. Dafou, C. Marth, E. Mueller-Holzner, M. Corcoran, M. Dagnell, et al. FBXW7/hCDC4 is a general tumor suppressor in human cancer. Cancer Res. 2007; 19:9006–12.
2. Garcia-Dios, D.A., D. Lambrechts, L. Coenegrachts, I. Vandenput, A. Capoen, P.M. Webb, K. Ferguson, Anecs, L.A. Akslen, B. Claes, I. Vergote, P. Moerman, J. Van Robays, et al. High-throughput interrogation of PIK3CA, PTEN, KRAS, FBXW7 and TP53 mutations in primary endometrial carcinoma. Gynecol Oncol. 2013; 2:327–34.
3. Sen, T., N. Sen, M.G. Noordhuis, R. Ravi, T.C. Wu, P.K. Ha, D. Sidransky, M.O. Hoque. OGDHL is a modifier of AKT-dependent signaling and NF-kappaB function. PLoS One. 2012; 11:e48770.
4. Zhang, Z., D. Alpert, R. Francis, B. Chatterjee, Q. Yu, T. Tansey, S.L. Sabol, C. Cui, Y. Bai, M. Koriabine, Y. Yoshinaga, J.F. Cheng, F. Chen, et al. Massively parallel sequencing identifies the gene Megf8 with ENU-induced mutation causing heterotaxy. Proc Natl Acad Sci U S A. 2009; 9:3219–24.
5. Wang, G.L., C.Y. Wang, X.Z. Cai, W. Chen, X.H. Wang, F. Li. Identification and expression analysis of a novel CW-type zinc finger protein MORC2 in cancer cells. Anat Rec (Hoboken). 2010; 6:1002–9.
6. Li, D.Q., S.S. Nair, K. Ohshiro, A. Kumar, V.S. Nair, S.B. Pakala, S.D. Reddy, R.P. Gajula, J. Eswaran, L. Aravind, R. Kumar. MORC2 signaling integrates phosphorylation-dependent, ATPase-coupled chromatin remodeling during the DNA damage response. Cell Rep. 2012; 6:1657–69.
7. Petrini, I., P.S. Meltzer, I.K. Kim, M. Lucchi, K.S. Park, G. Fontanini, J. Gao, P.A. Zucali, F. Calabrese, A. Favaretto, F. Rea, J. Rodriguez-Canales, R.L. Walker, et al. A specific missense mutation in GTF2I occurs at high frequency in thymic epithelial tumors. Nat Genet. 2014; 8:844–9.
